# *Fads2* knockout mice reveal that ALA prevention of hepatic steatosis is dependent on delta-6 desaturase activity

**DOI:** 10.1016/j.jlr.2024.100642

**Published:** 2024-09-19

**Authors:** Blair MacLeod, Chenxuan Wang, Liam H. Brown, Emma Borkowski, Manabu T. Nakamura, Kyle RD. Wells, Keith R. Brunt, Ewa Harasim-Symbor, Adrian Chabowski, David M. Mutch

**Affiliations:** 1 Department of Human Health and Nutritional Sciences, University of Guelph, Guelph, ON, Canada; 2 Department of Pathobiology, University of Guelph, Guelph, ON, Canada; 3 Department of Food Science and Human Nutrition, University of Illinois at Urbana-Champaign, Urbana, IL, USA; 4 Department of Pharmacology, Faculty of Medicine, Dalhousie University, Saint John, NB, Canada; 5 Department of Physiology, Medical University of Bialystok, Bialystok, Poland

**Keywords:** delta-6 desaturase, gene expression, glyceroneogenesis, *Fads2*, fish oil, lipogenesis, liver, omega-3 fatty acids, triacylglycerol

## Abstract

The production of the omega-3 long-chain polyunsaturated fatty acids (n-3 LCPUFA) eicosapentaenoic acid (EPA) and docosahexaenoic acid (DHA) from alpha-linolenic acid (ALA) relies on the delta-6 desaturase (D6D) enzyme encoded by the *Fads2* gene. While EPA and DHA reduce hepatic triacylglycerol (TAG) storage and regulate lipogenesis, the independent impact of ALA is less understood. To address this gap in knowledge, hepatic fatty acid metabolism was investigated in male wild-type (WT) and *Fads2* knockout (KO) mice fed diets (16% kcal from fat) containing either lard (no n-3 LCPUFA), flaxseed oil (ALA-rich), or menhaden oil (EPA/DHA rich) for 21 weeks. Fat content and composition, as well as markers of lipogenesis, glyceroneogenesis, and TAG synthesis, were analyzed using histology, gas chromatography, and reverse transcription quantitative PCR (RT-qPCR). Mice fed the menhaden diet had significantly lower hepatic TAG compared to both lard- and flax-fed mice, concomitant with changes in n-3 and n-6 LCPUFA in both TAG and phospholipid (PL) fractions (all *P* < 0.05). Flax-fed WT mice had lower liver TAG content compared to their KO counterparts. Menhaden-fed mice had significantly lower expression of key lipogenic (*Scd1, Srebp-1c, Fasn, Fads1, and Fads2*), glyceroneogenic (*Pck1*), and TAG synthesis (*Agpat3*) genes compared to lard, with flax-fed mice showing some intermediate effects. Gene expression effects were independent of D6D activity, since no differences were detected between WT and KO mice fed the same diet. This study demonstrates that EPA/DHA and not ALA itself is critical for the prevention of hepatic steatosis.

## Graphical Abstract

The graphical abstract was created with BioRender.com.

**Figure:**
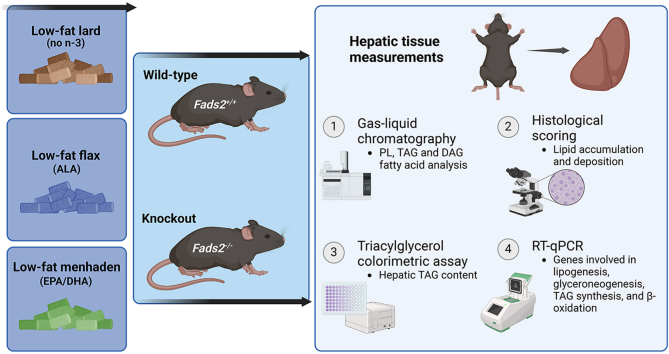

Perturbations in whole-body lipid metabolism are a common feature of conditions that are prevalent in Western society, such as metabolic dysfunction-associated steatotic liver disease (MASLD), type 2 diabetes, and obesity. The liver plays a central role in the regulation of whole-body lipid metabolism through various interconnected pathways including lipogenesis, glyceroneogenesis, triacylglycerol (TAG) production, and β-oxidation (1, 2). Dysfunction in these pathways can result in lipid accumulation and, consequently, hepatotoxicity and MASLD (3). Lifestyle interventions represent a cornerstone for the management and treatment of fatty liver disease (4). Increasing dietary omega-3 long-chain polyunsaturated fatty acid (n-3 LCPUFA) intake is a lifestyle intervention of particular interest due to their recognized TAG-lowering and anti-inflammatory properties, as well as their association with liver disease protection (5, 6).

The predominant dietary n-3 LCPUFA are alpha-linolenic acid (ALA), eicosapentaenoic acid (EPA), and docosahexaenoic acid (DHA). ALA is found mainly in plant oils such as flaxseed, soybean, and canola, while EPA and DHA are found in fatty fish and other seafood (7). Moreover, these n-3 LCPUFA are also present in several over-the-counter dietary supplements. However, a recent update to a worldwide n-3 LCPUFA status map revealed that blood EPA/DHA levels are still classified as being low to very low in many countries and regions (8). While there has been considerable research regarding the role of n-3 LCPUFA on the regulation of hepatic lipid metabolism, most research has focused on EPA and/or DHA. For example, EPA and DHA were shown to reduce hepatic TAG content in healthy mice (6), liver steatosis and inflammation in diet-induced obese mice (9), and hepatic lipogenesis in humans (10). Independent rodent studies have also shown that EPA/DHA downregulates the expression of key lipogenic genes such as *Srebp1* and *Scd1*, as well as *Fads1* and *Fads2* (11, 12, 13). Furthermore, mice fed a high-fat Western diet supplemented with n-3 LCPUFA had cholesterol levels similar to those observed in low-fat (10% total kcal) fed control animals (14). Finally, Feng *et al.* recently reported that increasing dietary EPA in TAG form from 2% to 6% caused a dose-dependent reduction in body weight, as well as liver lipid accumulation (15). Collectively, increased dietary EPA and DHA can improve liver lipid handling and prevent hepatic steatosis. In contrast, the independent effect of ALA on these processes is less understood.

In the liver, ALA can be converted into EPA and DHA, undergo β-oxidation for energy production, or be incorporated into TAG that is either stored locally or exported in VLDL (16). The first step in the conversion of ALA into EPA and DHA is mediated by the delta-6 desaturase (D6D), which is encoded by the *Fads2* gene (17, 18). Subsequent elongation (elongase two/five, ELOVL2/5) and desaturation (delta-5 desaturase, D5D) steps are necessary to produce EPA and DHA (19). Hepatic fat content was previously reported in humans to be associated with variants in genes influencing the efficiency of this conversion pathway, thereby suggesting that ALA and EPA/DHA may have distinct effects on hepatic lipid handling (20). As such, the development of a *Fads2* knockout model, which ablates the conversion of ALA into EPA and DHA, provides a valuable tool to distinguish the effects of different n-3 LCPUFA on metabolic outcomes (21).

The objective of this study was to investigate dietary n-3 LCPUFA regulation of hepatic steatosis and lipid metabolism. Specifically, we first examined the independent effects of dietary ALA versus EPA/DHA on markers of hepatic lipid metabolism. Using the *Fads2* KO mouse, we were also able to assess whether any effects observed with ALA on the regulation of hepatic lipid metabolism were independent of its conversion into downstream n3-LCPUFA such as EPA and DHA. Together, this study aimed to delineate the shared and distinct roles of ALA and EPA/DHA on the regulation of lipid metabolism in the liver.

## Materials and methods

### Animal care and feeding

All experimental protocols were approved by the University of Guelph Animal Care Committee in accordance with the requirements of the Canadian Council on Animal Care. Male and female heterozygous (*Fads2* ^+/−^) C57BL/6J mice were crossbred to generate the *Fads2*^*+/+*^ (WT) and *Fads2*^*−/−*^ (KO) mice used in this study. Mice in breeding harems were fed a modified AIN-93G diet containing corn oil as the primary source of fat (CAT# D03090904P, Research Diets, Inc). Male offspring were genotyped at 17 days of age via tail snipping to confirm the *Fads2* genotype. At 3 weeks of age, male mice were weaned and housed individually in shoebox cages in a temperature (22°C) and humidity-controlled room with a 12-h:12-h light:dark cycle. Mice were randomized to receive one of three isocaloric diets providing 16% kcal from fat (Table 1). The diets were modified from the standard AIN-93G diet to contain either 7% w/w lard (CAT# D16090607, Research Diets, Inc.), 7% w/w flaxseed oil (CAT# D12041404, Research Diets, Inc), or 7% w/w menhaden oil (CAT# D12041407, Research Diets, Inc), respectively. Lard and flax diets were supplemented with 0.37% (w/w) ARASCO oil (DSM Nutritional Products Ltd), which is rich in arachidonic acid (AA), to prevent side effects associated with an AA deficiency in KO mice (21) and to match the levels of AA naturally present in menhaden oil. Fatty acid content of the three diets is reported in supplemental Table S1. All mice had ad libitum access to their respective diets and water throughout the 21-week study. Body weight and food intake were measured weekly until the end of the study.

**Table 1:** Composition of experimental diets^a^

Nutritional Composition	Lard diet	Flax diet	Menhaden diet
Caloric content	kcal%		
Protein	20	20	20
Carbohydrate	64	64	64
Fat	16	16	16
kcal/g	4.0	4.0	4.0
Ingredient^b^	g/kg		
Casein	200	200	200
L-Cystine	3	3	3
Corn Starch	397.5	397.5	397.5
Maltodextrin 10	132	132	132
Sucrose	100	100	100
Cellulose, BW200	50	50	50
Lard	66.25	0	0
Flaxseed Oil	0	66.25	0
Menhaden Oil	0	0	70
ARASCO (40% ARA)	3.75	3.75	0
t-Butylhydroquinone	0.014	0.014	0.014
Mineral Mix^c^, S10022G	35	35	35
Vitamin Mix^c^, V10037	10	10	10
Choline Bitartrate	2.5	2.5	2.5

a Diets were prepared by Research Diets, Inc.

b Ingredients are expressed as g/kg.

c Composition details for the mineral (S10022G) and vitamin (V10037) mixes are available on the Research Diets Inc website.

### Tissue collection

At 24 weeks of age, mice were anesthetized with isoflurane followed by blood collection via cardiac puncture. Hepatic tissue was excised and weighed. A small piece of hepatic tissue was fixed in a 4% paraformaldehyde solution at 4°C for 24 h, and then washed with 1× PBS and stored in 70% ethanol at 4°C for histological processing. The remainder of the tissue was flash-frozen in liquid nitrogen immediately after collection and stored at −80°C until further analysis.

### Liver fatty acid analysis

The hepatic fatty acid composition was analyzed with gas chromatography-flame ionization detection (GC-FID). Briefly, hepatic lipids were extracted using a chloroform-methanol mixture (2:1, v/v) containing butylated hydroxytoluene and an internal standard (heptadecanoic acid). After incubating overnight, water was added, and samples were centrifuged for 10 min at 845 *g*. The lower layer was collected and subsequently separated using TLC on silica gel plates (Silica Plate 60, 0.25 mm; Merck) with a heptane/isopropyl ether/acetic acid (60:40:3, v/v) resolving solution. TAG, diacylglycerol (DAG), and phospholipid (PL) fractions were visualized under UV light and each fraction band was collected. Fractions were eluted using diethyl ether/hexane (1:1, v/v) and chloroform/methanol/water (5:5:1, v/v/v) solutions, and the organic phase was evaporated under a steady stream of nitrogen. Next, the procedure of transmethylation was conducted using a method developed by Christie (22). Briefly, a diethyl ether solution and methyl acetate were added to the samples containing either isolated TAG or PL fractions and mixed briefly. Under these conditions, lipids reacted with 1 M sodium methoxide in methanol for 10 min at room temperature. The reaction was stopped by adding a saturated solution of oxalic acid in diethyl ether. Samples were then mixed by vortex and then the solvent was evaporated under a steady stream of nitrogen. For the DAG fraction, we used boron trifluoride in a methanol solution for methylation and then incubated the samples for 10 min at 100°C. Pentane was used to extract fatty acid methyl esters, which was subsequently evaporated under a steady stream of nitrogen gas. Samples were then dissolved in hexane and analyzed with a Hewlett-Packard 5,890 Series II gas chromatograph, an Agilent J&W CP-Sil 88 capillary column (50m × 0.25 mm inner diameter), and a flame-ionization detector (Agilent Technologies). Fatty acid values are reported as total nmol fatty acid per g of liver tissue.

### Triacylglycerol colorimetric assay

Hepatic TAG content was measured with a Triglyceride Colorimetric Assay Kit (CAT#: 10010303, Cayman Chemical), as per the manufacturer's instructions. Tissue samples were first homogenized in 2 ml of NP40 Substitute Assay Reagent containing 10 μg/ml trypsin and centrifuged at 10,000 *g* for 10 min at 4°C. After centrifugation, the supernatant was collected and stored at −80°C until analysis. Glycerol levels were measured by a coupled enzymatic reaction system with a colorimetric readout at 540 nm. The absorbances of serially diluted TAG standards (0–200 mg/dl) were obtained and a standard curve was plotted to determine the TAG content of samples. TAG data is reported as mg/g liver tissue.

### Liver histology and steatosis scoring

Fixed hepatic tissues were placed in a tissue processor (CAT#: A78400111, Shandon Excelsior ES®, Thermo Scientific), which performed automatic 45-min submersions in increasing isopropanol concentrations at 70%, 85%, 90%, 95%, and 100% under vacuum conditions, followed by three 45-min submersion steps in xylene and three 45-min submersion steps in paraffin wax. Tissues were embedded in paraffin wax on an embedding workstation (CAT#: A81000101, HistoStarTM, Thermo Scientific). Embedded tissue blocks were sectioned at 5.0 μm using a rotary microtome (CAT#: RM2255, Leica Bio-systems). Sections were allowed to smoothen in a water bath before transfer to microscope slides (CAT#: 3800050, Leica Bio-systems, Deer Park, IL). Slides were dried and fixed overnight on a 37°C hot plate. Hematoxylin and eosin (H&E) staining was performed by submerging the fixed slides in xylene three times for 2 min each, followed by three 2-min submersion steps in 100% isopropanol, a 2-min submersion step in 70% isopropanol, and a 2-min submersion step in deionized water. Next, slides were transferred to Harris Modified Hematoxylin stain with acetic acid for 10 min, followed by thorough rinsing in deionized water. The slides were then dipped eight times in an acid alcohol solution (70% isopropanol and 1% hydrochloric acid). After a thorough wash in deionized water, the slides were dipped in ammonia water until the tissue turned blue, followed by another washing step in deionized water. Next, slides were dipped six times in 70% isopropanol before being placed in an eosin staining solution (0.2% eosin Y solution, 60% ethanol, and 0.5% glacial acetic acid) for 30 s, followed by a dehydration step via three 2-min 100% isopropanol submersions and a clear step via three 2-min xylene submersions. Finally, coverslips were placed over tissue sections using CytosealTM XYL mounting medium (CAT#: 8312-16E, Thermo Scientific) and dried overnight in a fume hood. Slides were scanned using a Panoramic MIDI 3D Histech Slide Scanner. Light microscopy was used to evaluate hepatic steatosis using a clinical fatty liver disease activity score (23). Samples were independently scored by three individuals and the score was averaged for each sample. A score of 0–3 was assigned to each sample, where 0 = <5%; 1 = 5%–33%; 2 = 33%–66%; 3 = >66% fat accumulation.

### RNA extraction and gene expression analysis

Total RNA from liver samples was extracted using TRIzol Reagent for tissue homogenization (CAT#:15596026, Invitrogen, Waltham, MA) and the RNeasy Mini Kit (CAT#: 74106, Qiagen), as per manufacturer's instructions. RNA concentration and purity were measured using a NanoDrop™ 2000 Spectrophotometer (Thermo Fisher Scientific, Wilmington, DE). cDNA was synthesized from 1 μg of total RNA using the Applied Biosystems™ High-Capacity cDNA Reverse Transcription Kit (CAT#: 4368814, Thermo Fisher Scientific Wilmington, DE), as per manufacturer's instructions. Primers for *Fads1*, *Fads2*, *Dgat1*, *Dgat2*, *Agpat3, Ppar-α, Cpt1a, Srebp-1c, Scd1*, *Fasn*, *Acc*, *Pck-1*, *Gk*, *and Aqp9* were designed using NCBI Primer-BLAST software (24). Primer sequences are listed in supplemental Table S2. Reverse transcription-quantitative PCR (RT-qPCR) was performed as previously described (25). Briefly, the total reaction volume per sample was 10 μl, which consisted of 5 μl SsoFast Evagreen Supermix (CAT#: 1725204, Bio-Rad Laboratories), 2.5 μl cDNA, 0.2 μl of 10 μM forward and reverse primer mixture, and 2.3 μl nuclease-free water. RT-qPCR was run using a Bio-Rad CFX-96 Real-Time system with the following cycling conditions: one denaturing cycle at 95°C for 30 s, followed by 40 cycles of 95°C for 4 s and 55.9°C for 4 s *18s* was used as a reference gene for normalization. Data was analyzed using the ΔΔCt method.

### Statistical analysis

Data were analyzed using either GraphPad Prism, Version 10.2.0 (GraphPad Software Incorporated, La Jolla, CA) or R, Version 4.0.2 (R Core Team, 2021). Fatty acid and RT-qPCR data were analyzed with GraphPad Prism using a two-way ANOVA for the main effects of diet (*P*_*D*_) and genotype (*P*_*G*_), as well as their interaction (*P*_*I*_), followed by a *post-hoc* Tukey test for multiple comparisons. Food intake and body weight gain during the feeding study were analyzed with R using a three-way ANOVA for the main effects of time (not shown), diet, and genotype as well as the diet × genotype interaction, followed by a *post hoc* Tukey HSD test for multiple comparisons. A *P* ≤ 0.05 was used as the threshold for statistical significance. Results are presented as mean ± standard error mean (SEM).

## Results

### N-3 LCPUFA do not affect food intake, feed efficiency, body or liver weight

**Fig. 1:**
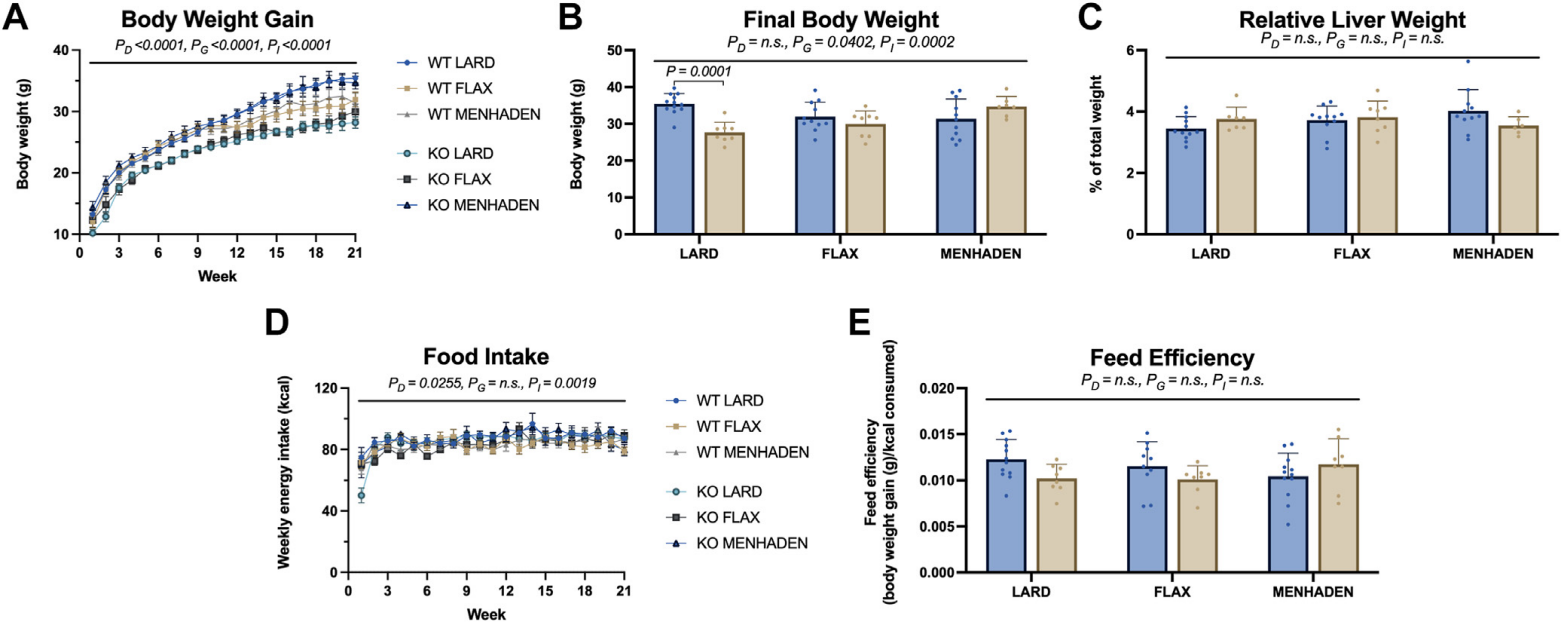
Anthropomorphic measurements and food intake/efficiency show minor differences with ALA or EPA/DHA feeding. Graphs for (A) body weight gain, (B) final body weight, (C) relative liver weight, (D) food intake and (E) feed efficiency are shown. Statistical significance (main effect; diet (*P*_*D*_), genotype (*P*_*G*_), and interaction (*P*_*I*_)) is indicated with a horizontal line accompanied by the associated *P*-value (significance *P* < 0.05). Data are reported as mean ± standard error mean (SEM). N = 8–12 mice/group.

A main effect of genotype and diet, as well as an interaction between these main effects, was found for body weight gain (Fig. 1A; all *P* < 0.0001). When considering final body weight (Fig. 1B), a significant interaction was detected (*P*_*I*_ = 0.0002) that was primarily driven by a genotype difference showing that lard-fed WT mice weighed more than lard-fed KO mice (*P* = 0.0001, Fig. 1B). Diet did not have a significant effect on final body weight (Fig. 1B). Relative liver weight was not affected by either diet or genotype (Fig. 1C). A small effect of diet was observed on food intake (Fig. 1D; *P*_*D*_ = 0.0255), with no differences observed in feed efficiency (Fig. 1E).

### N-3 and n-6 LCPUFA composition is altered by ALA and EPA/DHA

**Fig. 2:**
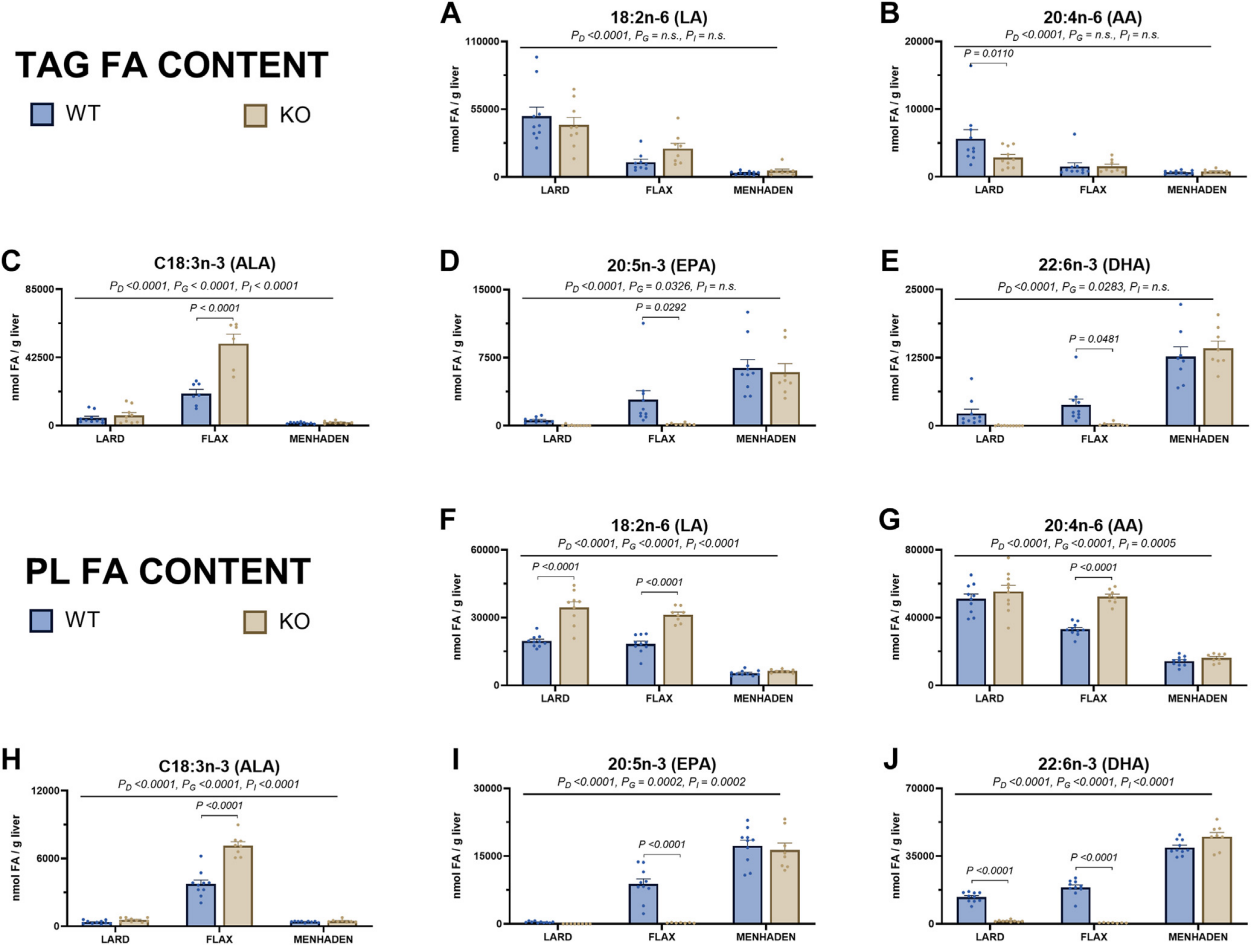
N-3 and n-6 PUFA concentrations are significantly altered with ALA and EPA/DHA feeding. Graphs for key n-3 and n-6 PUFA concentrations within TAG (A–E) and PL (F–J) fractions are shown. Statistical significance for the 2-way ANOVA (main effect; diet (*P*_*D*_), genotype (*P*_*G*_), and interaction (*P*_*I*_)) is indicated with a horizontal line accompanied by the associated *P*-value (significance *P* < 0.05). Post-hoc tests showing a significant difference between wildtype (WT) and knockout (KO) mice within a diet group are also indicated. Data are reported as mean ± standard error mean (SEM). N = 8–12 mice/group.

The n-3 and n-6 LCPUFA species present in hepatic TAG and PL fractions were consistently altered with diet and genotype (Fig. 2A–J and supplemental Table S3). As expected, WT and KO mice fed the menhaden diet had higher EPA and DHA content compared to lard- and flax-fed mice (*P*_*D*_ < 0.0001, Fig. 2D, E), while flax-fed mice had higher ALA content compared to lard- and menhaden-fed mice (*P*_*D*_ < 0.0001, Fig. 2C), in both TAG and PL fractions. Post-hoc analysis revealed that flax-fed KO mice had higher ALA (*P* < 0.0001, Fig. 2C) concomitant with lower EPA and DHA compared to their WT counterparts (both *P* < 0.05, Fig. 2D, E). A similar pattern was observed for LA content in the PL fraction of lard- and flax-fed mice, but not menhaden-fed mice (Fig. 2A, F). While LA content in TAG was significantly lower in menhaden-fed mice compared to lard- and flax-fed mice, no genotype effect was observed. Similarly, AA content was lower in menhaden-fed mice in both the TAG and PL fractions, with no effect of genotype (Fig. 2B, G).

### ALA and EPA/DHA reduce total TAG and DAG content and alter hepatic steatosis

**Fig. 3:**
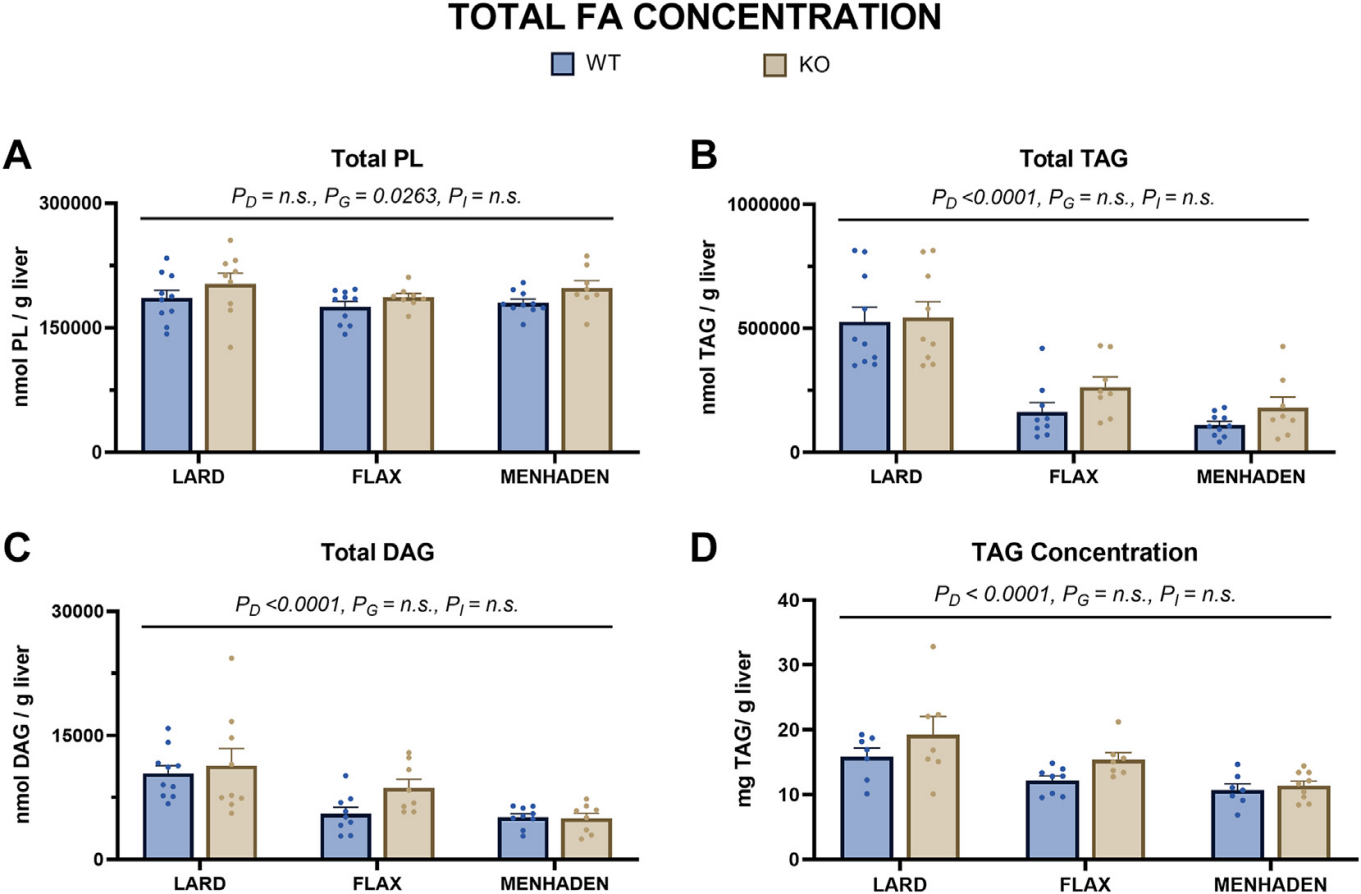
ALA and EPA/DHA feeding alter hepatic fatty acid (FA) concentration. Graphs for total FA concentration in (A) phospholipid (PL), (B) triacylglycerol (TAG) and (C) diacylglycerol (DAG) fractions along with (D) TAG concentration via the TAG colorimetric assay are shown. Statistical significance for the 2-way ANOVA (main effect; diet (*P*_*D*_), genotype (*P*_*G*_), and interaction (*P*_*I*_)) is indicated with a horizontal line accompanied by the associated *P*-value (significance *P* < 0.05). Data are reported as mean ± standard error mean (SEM). N = 8–12 mice/group.

**Fig. 4:**
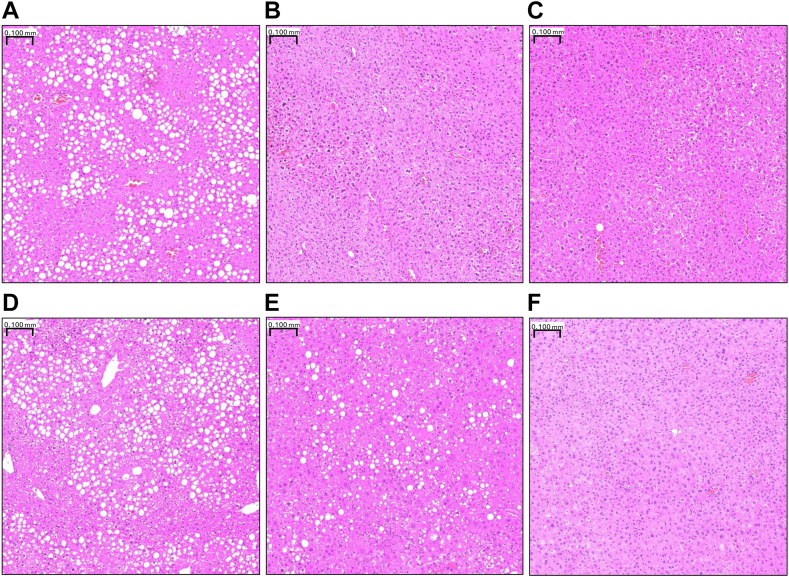
Hepatic steatosis is differentially altered by ALA or EPA/DHA feeding. Representative H&E stained liver sections for (A) wildtype (WT) lard, (B) WT flax, (C) WT menhaden, (D) knockout (KO) lard, (E) KO flax, and (F) KO menhaden mice are shown. Magnification 10×.

Fatty acids within each lipid fraction were summed to estimate total PL, TAG, and DAG content (Fig. 3). No major differences were detected in estimated total PL between groups (Fig. 3A). In contrast, both flax- and menhaden-fed mice showed lower estimated total TAG compared to lard-fed mice (*P*_D_ < 0.0001, Fig. 3B), regardless of genotype. Similarly, both flax- and menhaden-fed mice had lower estimated total DAG compared to lard-fed mice, regardless of genotype (*P*_D_ < 0.0001, Fig. 3C). The differences in the estimated total TAG content were subsequently confirmed using a colorimetric assay (Fig. 3D; Flax [*P* = 0.0082] and menhaden [*P* < 0.0001] vs. lard fed mice). No statistically significant differences were observed between genotypes within a diet group. Lard-fed WT and KO mice had the highest steatosis scores relative to other diet groups (Table 2 and Fig. 4A–F). Flax-fed KO mice had a steatosis score like that seen in lard-fed mice, while flax-fed WT mice had a score that was more comparable with that observed in menhaden-fed mice. Neither WT nor KO menhaden-fed mice showed signs of steatosis (Fig. 4C, F).

**Table 2:** Liver steatosis scores after 21 weeks of diet

Treatment Group	Steatosis Score^a^
Lard WT	1.3
Flax WT	0
Menh WT	0
Lard KO	0.9
Flax KO	0.8
Menh KO	0

a An arbitrary score of 0–3 was assigned for steatosis (0 = <5%; 1 = 5%–33%; 2 = 33%–66%; 3 = >66%). Values are presented as the mean steatosis score for each group.

### EPA/DHA but not ALA lower hepatic lipogenic gene expression

**Fig. 5:**
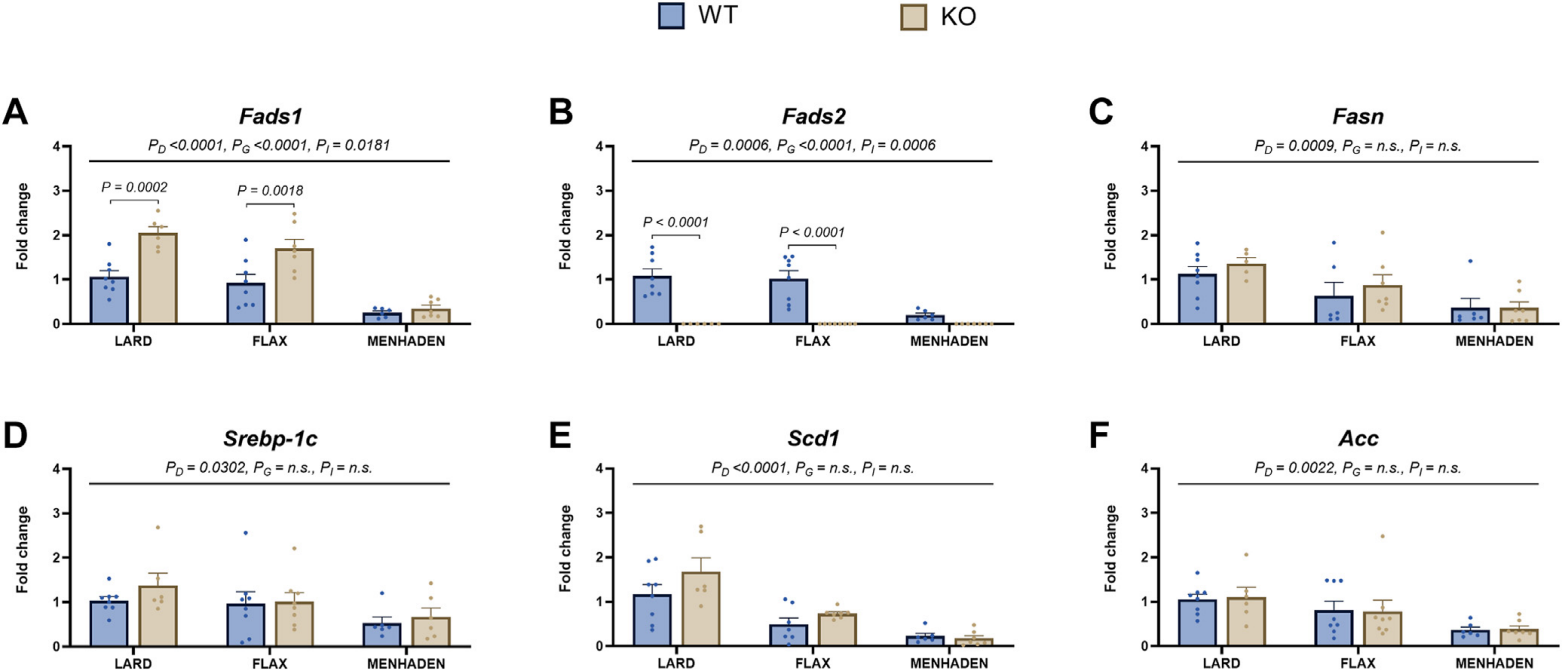
Lipogenic gene expression is lower with EPA/DHA feeding. Graphs for hepatic expression of (A) *Fads1*, (B) *Fads2*, (C) *Fasn*, (D) *Srebp-1c*, (E) *Scd1* and (F) *Acc* are shown. Statistical significance for the 2-way ANOVA (main effect; diet (*P*_*D*_), genotype (*P*_*G*_), and interaction (*P*_*I*_)) is indicated with a horizontal line accompanied by the associated *P*-value (significance *P* < 0.05). Post-hoc tests showing a significant difference between wildtype (WT) and knockout (KO) mice within a diet group are also indicated. Data are reported as mean ± standard error mean (SEM). N = 6–8 mice/group. Fold change is relative to WT lard-fed control mice.

We next examined a panel of genes that regulate lipogenesis to determine if differences in TAG content between dietary groups stemmed from underlying changes in gene expression. The expression of *Fads1, Fads2, Fasn, Srebp-1c, Scd1*, and *Acc* were all significantly lower in menhaden-fed mice compared to the other diet groups (all *P*_*D*_ < 0.05, Fig. 5A–F), with little-to-no differences noted between lard- and flax-fed mice apart from the expression of *Scd1* (*P*_*D*_ < 0.0001, Fig. 5E). Specifically, *Scd1* expression in flax-fed mice was intermediate to lard- and menhaden-fed mice. Post-hoc analysis showed significant increases in *Fads1* expression were observed in KO compared to WT mice fed lard and flax diets (*P* = 0.0002 and *P* = 0.0018, respectively; Fig. 5A). Furthermore, post hoc analysis confirmed that *Fads2* expression was ablated in KO compared to WT mice fed either lard or flax diets (*P* < 0.0001, Fig. 5B). *Fads2* expression in WT menhaden-fed mice was considerably lower than that seen in lard- and flax-fed WT mice.

### N-3 PUFA alters the expression of key hepatic lipid metabolism genes

**Fig. 6:**
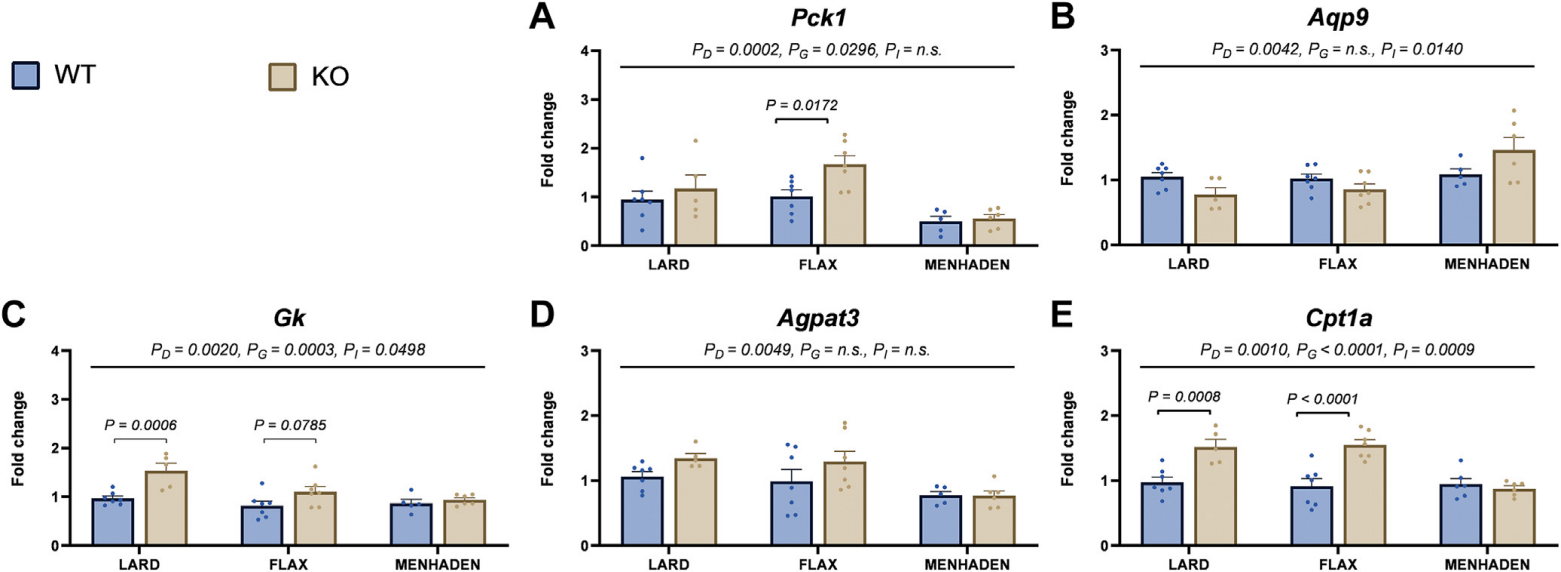
EPA/DHA feeding alters hepatic expression of lipid metabolism genes. Graphs for hepatic expression of key genes involved in (A–C) glyceroneogenesis, (D) triacylglycerol (TAG) synthesis, and (E) β-oxidation are shown. Statistical significance for the 2-way ANOVA ((main effect; diet (*P*_*D*_), genotype (*P*_*G*_), and interaction (*P*_*I*_)) is indicated with a horizontal line accompanied by the associated *P*-value (significance *P* < 0.05). Post-hoc tests showing a significant difference between wildtype (WT) and knockout (KO) mice within a diet group are also indicated. Data are reported as mean ± standard error mean (SEM). N = 6–8 mice/group. Fold change is relative to WT lard-fed control mice.

Significant effects on the expression of genes involved in glyceroneogenesis, TAG synthesis, and β-oxidation were also observed with diet (*P*_*D*_ < 0.001). Post hoc analysis showed that menhaden-fed mice had lower *Pck1* expression compared to both lard- and flax-fed mice (*P* = 0.0148 and *P* < 0.0001, respectively, Fig. 6A) and higher *Aqp9* expression (*P* = 0.0089 and *P* = 0.0114, respectively, Fig. 6B). Furthermore, *Pck1* expression was higher in KO versus WT flax-fed mice (*P* = 0.0172, Fig. 6A). *Gk* showed a similar pattern of expression between WT and KO mice in the lard and flax groups, but not menhaden (Fig. 6C). *Agpat3* expression was significantly lower in menhaden-fed mice compared to both lard and flax-fed mice (*P* = 0.0074 and *P* = 0.0195, respectively, Fig. 6D). *Cpt1a* expression was higher in KO compared to WT mice fed lard and flax diets (*P* = 0.0008 and *P* < 0.0001, respectively, Fig. 6E), with no difference observed between genotypes in menhaden-fed animals.

## Discussion

Although the beneficial role of EPA and DHA on hepatic steatosis are well described in the literature, the independent effects of ALA are less clear. Consequently, we aimed to address this gap in knowledge by investigating the impact of dietary ALA versus EPA/DHA on hepatic lipid metabolism using the *Fads2* KO mouse model. Our findings show that dietary EPA/DHA can suppress hepatic lipid metabolism by preventing TAG accumulation, reducing lipogenic gene expression, and regulating various markers of hepatic glyceroneogenesis and β-oxidation. While dietary ALA promoted intermediate trends in several of the measured outcomes, these effects were not observed when ALA conversion to EPA/DHA was prevented in *Fads2* KO mice. Collectively, this suggests that ALA conversion into EPA/DHA is necessary to prevent steatosis and regulate lipid handling pathways in the liver.

Both flax- and menhaden-fed mice had lower TAG and DAG content compared to lard-fed mice, with the prevention of TAG deposition being more pronounced in menhaden-fed mice. This points to the ability of n-3 LCPUFA to reduce hepatic lipid content, a finding that is in agreement with recent literature showing that EPA and DHA feeding results in lower hepatic TAG compared to high-fat-fed controls in murine models (26, 27). Through post-hoc analysis, we found that TAG content in flax-fed WT mice was intermediate to that observed in lard- and menhaden-fed mice. In contrast, a trend (*P* = 0.09) for higher liver TAG content was noted between flax-fed KO mice and menhaden-fed mice, which aligned with histological scoring of liver lipid content. Together, these results insinuate a greater risk for hepatic steatosis in the absence of either endogenously synthesized or exogenously supplied EPA and DHA. Importantly, our results align with a study by Wang *et al.* who reported that humans carrying rare alleles in the *FADS1* gene, which are associated with lower endogenous synthesis of EPA and DHA compared to individuals carrying the common allele, had higher liver fat content (20).

As expected, hepatic fatty acid content reflected dietary fatty acid intake, ie, higher ALA in flax-fed mice and higher EPA/DHA in menhaden-fed mice. Moreover, the absence of liver EPA and DHA in flax-fed KO mice, concomitant with the increase in ALA, substantiated the ablation of D6D activity. It is notable that flax-fed WT mice, which can convert ALA into downstream n-3 LCPUFA, did not achieve comparable hepatic levels of EPA and DHA compared to those seen in menhaden-fed mice in either the TAG or PL fractions. We also observed lower LA and AA in both the TAG and PL fractions of menhaden-fed mice, regardless of genotype. While the low LA most likely stems from its low abundance in the menhaden diet (supplemental Table S1), our results for AA suggest a more biological explanation since AA levels were matched across the three experimental diets. Indeed, our findings suggest that both endogenously produced EPA and DHA, as well as dietary EPA and DHA, may be displacing AA in PL, and to a lesser extent TAG. These findings were not observed in flax-fed KO mice, highlighting that EPA/DHA are incorporated into the hepatic PL pool, while ALA is predominantly incorporated into the hepatic TAG pool.

Histological evaluation revealed a reduction in hepatic steatosis in menhaden-fed mice compared to lard-fed mice, with flax-fed mice showing an effect that was dependent on the *Fads2* genotype. Flax-fed KO mice had steatosis scores like lard-fed mice, while flax-fed WT mice had steatosis scores that aligned with menhaden-fed mice. This suggests that the presence of EPA and DHA is necessary to prevent hepatic lipid deposition. This corresponds with a prior report where mice fed diets containing high ALA had higher levels of EPA and DHA in the liver and, subsequently, lower steatosis in comparison with mice fed diets with lower amounts of ALA (13). This dose-response effect further suggests that any benefits on hepatic steatosis observed with dietary ALA stem from the production of EPA and DHA rather than ALA itself (28). In contrast, our results don’t fully agree with a prior study in which liver lipid accumulation was examined in WT and KO *Fads2* mice fed lard, flax, and menhaden diets (9). While menhaden-fed mice in this prior study also had the lowest hepatic deposition, no difference in steatosis was observed between WT and KO flax-fed mice. However, an important distinction is that Monteiro *et al.* fed mice diets providing 30% kcal from fat compared to the 16% used in the present study. It is therefore plausible that the higher overall fat content masked the genotype effect.

We also found that ALA had a limited effect on the expression of key hepatic lipogenic genes in flax-fed KO mice. Interestingly, flax-fed WT mice also did not show significant changes in lipogenic gene expression, suggesting that the amount of EPA and DHA endogenously produced in these mice may have been insufficient to regulate the expression of these genes. In contrast, menhaden-fed mice showed significantly lower expression of all lipogenic genes examined (*Fads1, Fads2, Fasn, Srebp-1c, Scd1*, and *Acc*) compared to the other diet groups. Further, menhaden-fed WT mice showed a strong down-regulation in *Fads2* expression, while both WT and KO mice showed a strong down-regulation in *Fads1* expression. This reinforces the feedback inhibition of dietary EPA and DHA on genes regulating the desaturation pathway (29, 30). We also noted a distinct regulation by different n-3 LCPUFA on the expression of genes involved in glyceroneogeneis, TAG synthesis, and β-oxidation pathways. For example, mice fed the menhaden diet had lower *Pck1* and *Agpat3* expression, as well as an increase in *Aqp9* expression, compared to both lard- and flax-fed mice. Neither *Cpt1a* nor *Gk* expression was impacted by the menhaden diet. Interestingly, *Pck1*, *Gk,* and *Cpt1a* expression were all increased in lard- and flax-fed KO mice compared to their WT counterparts. This is notable since we recently showed that reduced D6D activity in these KO mice caused a reduction in white adipose tissue TAG content by inhibiting fatty acid re-esterification (25). As such, it is plausible that the increase in hepatic expression of *Pck1*, *Gk*, and *Cpt1a* in *Fads2* KO mice highlights compensatory responses in TAG production and β-oxidation to help the liver deal with a greater influx of non-esterified fatty acids (NEFA) from adipose tissue. In contrast, KO mice fed the menhaden diet do not show these changes compared to their WT counterparts, suggesting that EPA and DHA provide a buffer against perturbations in whole-body lipid handling associated with reduced D6D activity.

Our work may initially appear to partially contrast with previous literature demonstrating that mice fed ALA-containing diets have lower expression and activity of key hepatic lipid metabolism genes and enzymes (31, 32, 33); however, an important caveat when interpreting results from past research is the inability to attribute outcomes to ALA specifically versus the endogenously produced downstream n-3 LCPUFA, such as EPA and DHA. Further, it is plausible that higher amounts of ALA (which subsequently leads to higher endogenous production of downstream n-3 LCPUFA) may be necessary to elicit an effect (31). This is an important point of consideration given that the flax-fed WT mice in the present study did not achieve liver concentrations of EPA and DHA in either PL or TAG fractions comparable to those seen in menhaden-fed mice. This underscores potential differences in the hepatic accretion of EPA and DHA generated through endogenous conversion versus exogenous consumption (34). With regard to the endogenous conversion of ALA into EPA and DHA, past tracer studies have suggested that ∼6% and ∼1% of ALA is converted into EPA and DHA, respectively, based on the analysis of blood fractions (16, 35). Although recent studies using compound-specific isotope analysis (CSIA) suggest these historical estimates may underestimate actual conversion rates when factoring in whole-body EPA and DHA synthesis (36), the majority of dietary ALA is partitioned towards storage in tissues (eg, adipose tissue) or β-oxidation (37). Further, we recognize that a limitation of the current study is our inability to ascertain whether the effects seen are due to either EPA alone, DHA alone, or the combination. Moreover, we cannot exclude that the effects observed may in part be due to other n-3 LCPUFA in the pathway, notably n-3 docosapentaenoic acid (DPAn-3) (38).

We acknowledge that additional limitations exist with our study. First, it is possible that some of the modest effects seen at the gene expression level may not be reflected at the protein level. Therefore, future studies in the area should include protein measurements and/or functional assays (eg, hepatic microsomes). Second, the use of dietary oils rather than purified fatty acids means that the experimental diets differed regarding saturated, monounsaturated and n-6 LCPUFA composition. For example, LA levels were similar between the lard and flax diets, but considerably lower in the menhaden diet, and saturated fats were similar between lard and menhaden diets but much lower in the flax diet. However, the addition of ARASCO to the lard and flax diets allowed our diets to be comparable with respect to AA content. When designing the experimental diets, our primary focus was on the n-3 LCPUFA content of the dietary oils rather than the other fatty acids. Nevertheless, we can not exclude an impact for differences in other fatty acids on our study outcomes. Thus, future research using purified n-3 LCPUFA diets may provide additional insights into their shared and unique roles on hepatic lipid metabolism. Finally, we are cognizant that the translatability of our findings to human health remains uncertain when it comes to the diet effects. While valuable for elucidating specific metabolic outcomes, the high amounts of ALA and EPA/DHA found in the flax and menhaden diets, respectively, are not reflective of a typical human diet. With this said, it is notable that the hepatic phenotype observed in flax-fed WT and KO mice aligns with that previously reported in humans carrying genetic variants affecting the efficiency of the desaturase pathway (21). Future studies using different doses of n-3 LCPUFA will help address this limitation and potentially improve translatability to humans.

## Conclusion and future research

The present study demonstrated that the consumption of a diet supplemented with EPA and DHA prevented hepatic steatosis through the inhibition of lipogenic gene expression compared to a diet rich in ALA. Additionally, we show that ALA conversion into downstream n-3 LCPUFA is essential to prevent hepatic lipid deposition; however, the overall effect on study outcomes in flax-fed WT mice was less significant than those observed in menhaden-fed mice. These findings help to advance the understanding of the distinct effects of different n-3 LCPUFA on hepatic lipid metabolism and suggest that the benefits of ALA on hepatic lipogenesis and lipid deposition stem from downstream n-3 LCPUFA rather than ALA itself. Further research aimed at elucidating the distinct effects of these n-3 LCPUFA in relation to the whole-body handling of fatty acids will further clarify the roles of individual n-3 LCPUFA on systemic lipid metabolism, particularly in the context of MASLD.

## Data availability

The datasets generated and analyzed for the current study are available from the corresponding author on reasonable request. All data generated and analyzed during this study are included in this published article.

## Supplemental data

This article contains supplemental data.

## Conflict of interest

The authors declare that they have no conflicts of interest with the contents of this article.

## References

[1] Trefts E., Gannon M., Wasserman D.H. (2017). The liver. Curr. Biol..

[2] Heeren J., Scheja L. (2021). Metabolic-associated fatty liver disease and lipoprotein metabolism. Mol. Metab..

[3] Syed-Abdul M.M. (2023). Lipid metabolism in metabolic-associated steatotic liver disease (MASLD). Metabolites.

[4] Younossi Z.M., Zelber-Sagi S., Henry L., Gerber L.H. (2023). Lifestyle interventions in nonalcoholic fatty liver disease. Nat. Rev. Gastroenterol. Hepatol..

[5] Vell M.S., Creasy K.T., Scorletti E., Seeling K.S., Hehl L., Rendel M.D. (2023). Omega-3 intake is associated with liver disease protection. Front. Public Health.

[6] Rajna A., Brown L.H., Frangos S.M., Gonzalez-Soto M., Hucik B., Wang C. (2022). Plant and marine N3-PUFA regulation of fatty acid trafficking along the adipose tissue-liver axis varies according to nutritional state. J. Nutr. Biochem..

[7] Surette M.E. (2008). The science behind dietary omega-3 fatty acids. Can. Med. Assoc. J..

[8] Schuchardt J.P., Beinhorn P., Hu X.F., Chan H.M., Roke K., Bernasconi A. (2024). Omega-3 world map: 2024 update. Prog. Lipid Res..

[9] Monteiro J., Askarian F., Nakamura M.T., Moghadasian M.H., Ma D.W.L. (2013). Oils rich in α-linolenic acid independently protect against characteristics of fatty liver disease in the Δ6-desaturase null mouse. Can. J. Physiol. Pharmacol..

[10] Green C.J., Pramfalk C., Charlton C.A., Gunn P.J., Cornfield T., Pavlides M. (2020). Hepatic de novo lipogenesis is suppressed and fat oxidation is increased by omega-3 fatty acids at the expense of glucose metabolism. BMJ Open Diab Res. Care.

[11] Gillies P.J., Bhatia S.K., Belcher L.A., Hannon D.B., Thompson J.T., Vanden Heuvel J.P. (2012). Regulation of inflammatory and lipid metabolism genes by eicosapentaenoic acid-rich oil. J. Lipid Res..

[12] Sampath H., Miyazaki M., Dobrzyn A., Ntambi J.M. (2007). Stearoyl-CoA desaturase-1 mediates the pro-lipogenic effects of dietary saturated fat. J. Biol. Chem..

[13] Endo Y., Onodera A., Obata-Ninomiya K., Koyama-Nasu R., Asou H.K., Ito T. (2019). ACC1 determines memory potential of individual CD4+ T cells by regulating de novo fatty acid biosynthesis. Nat. Metab..

[14] Yuan F., Wang H., Tian Y., Li Q., He L., Li N. (2016). Fish oil alleviated high-fat diet–induced non-alcoholic fatty liver disease via regulating hepatic lipids metabolism and metaflammation: a transcriptomic study. Lipids Health Dis..

[15] Feng J., Wang S., Chen F., Zhang J., Wang Q., Jiang L. (2024). Effects of triglyceride and ethyl ester forms of EPA on hepatic lipid metabolism in mice with non-alcoholic fatty liver disease. J. Funct. Foods.

[16] Burdge G.C. (2006). Metabolism of α-linolenic acid in humans. Prostaglandins Leukot. Essent. Fatty Acids.

[17] Li Y.L., Tian H., Jiang J., Zhang Y., Qi X.W. (2020). Multifaceted regulation and functions of fatty acid desaturase 2 in human cancers. Am. J. Cancer Res..

[18] Vaittinen M., Walle P., Kuosmanen E., Männistö V., Käkelä P., Ågren J. (2016). FADS2 genotype regulates delta-6 desaturase activity and inflammation in human adipose tissue. J. Lipid Res..

[19] Ratnayake W.M.N., Galli C. (2009). Fat and fatty acid terminology, methods of analysis and fat digestion and metabolism: a background review paper. Ann. Nutr. Metab..

[20] Wang L., Athinarayanan S., Jiang G., Chalasani N., Zhang M., Liu W. (2015). Fatty acid desaturase 1 gene polymorphisms control human hepatic lipid composition. Hepatology.

[21] Stroud C.K., Nara T.Y., Roqueta-Rivera M., Radlowski E.C., Lawrence P., Zhang Y. (2009). Disruption of FADS2 gene in mice impairs male reproduction and causes dermal and intestinal ulceration. J. Lipid Res..

[22] Christie W.W. (1982). A simple procedure for rapid transmethylation of glycerolipids and cholesteryl esters. J. Lipid Res..

[23] Brunt E.M., Kleiner D.E., Wilson L.A., Belt P., Neuschwander-Tetri B.A. (2011). For the NASH Clinical Research Network (CRN). Nonalcoholic fatty liver disease (NAFLD) activity score and the histopathologic diagnosis in NAFLD: distinct clinicopathologic meanings. Hepatology.

[24] Ye J., Coulouris G., Zaretskaya I., Cutcutache I., Rozen S., Madden T.L. (2012). Primer-BLAST: a tool to design target-specific primers for polymerase chain reaction. BMC Bioinformatics.

[25] Wang C., Hucik B., Sarr O., Brown L.H., Wells K.R.D., Brunt K.R. (2023). Delta-6 desaturase (Fads2) deficiency alters triacylglycerol/fatty acid cycling in murine white adipose tissue. J. Lipid Res..

[26] Gao J., Xiao H., Li J., Guo X., Cai W., Li D. (2019). N-3 polyunsaturated fatty acids decrease long-term diabetic risk of offspring of gestational diabetes rats by postponing shortening of hepatic telomeres and modulating liver metabolism. Nutrients.

[27] Shang T., Liu L., Zhou J., Zhang M., Hu Q., Fang M. (2017). Protective effects of various ratios of DHA/EPA supplementation on high-fat diet-induced liver damage in mice. Lipids Health Dis..

[28] Kelley D.S., Nelson G.J., Love J.E., Branch L.B., Taylor P.C., Schmidt P.C. (1993). Dietary α-linolenic acid alters tissue fatty acid composition, but not blood lipids, lipoproteins or coagulation status in humans. Lipids.

[29] Tanaka S., Ishihara N., Suzuki S., Watanabe Y., Nagayama D., Yamaguchi T. (2019). Fatty acid desaturase 2 is up-regulated by the treatment with statin through geranylgeranyl pyrophosphate-dependent Rho kinase pathway in HepG2 cells. Sci. Rep..

[30] Metherel A.H., Valenzuela R., Klievik B.J., Cisbani G., Rotarescu R.D., Gonzalez-Soto M. (2024). Dietary docosahexaenoic acid (DHA) downregulates liver DHA synthesis by inhibiting eicosapentaenoic acid elongation. J. Lipid Res..

[31] Rincón-Cervera M.Á., Valenzuela R., Hernandez-Rodas M.C., Barrera C., Espinosa A., Marambio M. (2016). Vegetable oils rich in alpha linolenic acid increment hepatic n-3 LCPUFA, modulating the fatty acid metabolism and antioxidant response in rats. Prostaglandins Leukot. Essent. Fatty Acids.

[32] Su J., Ma C., Liu C., Gao C., Nie R., Wang H. (2016). Hypolipidemic activity of peony seed oil rich in α-linolenic, is mediated through inhibition of lipogenesis and upregulation of fatty acid β-oxidation. J. Food Sci..

[33] De Tonnac A., Labussière E., Vincent A., Mourot J. (2016). Effect of *α* -linolenic acid and DHA intake on lipogenesis and gene expression involved in fatty acid metabolism in growing-finishing pigs. Br. J. Nutr..

[34] Austin G.L., Ogden L.G., Hill J.O. (2011). Trends in carbohydrate, fat, and protein intakes and association with energy intake in normal-weight, overweight, and obese individuals: 1971–2006. Am. J. Clin. Nutr..

[35] Plourde M., Cunnane S.C. (2007). Extremely limited synthesis of long chain polyunsaturates in adults: implications for their dietary essentiality and use as supplements. Appl. Physiol. Nutr. Metab..

[36] Rotarescu R.D., Mathur M., Bejoy A.M., Anderson G.H., Metherel A.H. (2024). Serum measures of docosahexaenoic acid (DHA) synthesis underestimates whole body DHA synthesis in male and female mice. J. Nutr. Biochem..

[37] McCloy U., Ryan M.A., Pencharz P.B., Ross R.J., Cunnane S.C. (2004). A comparison of the metabolism of eighteen-carbon 13C-unsaturated fatty acids in healthy women. J. Lipid Res..

[38] Drouin G., Rioux V., Legrand P. (2019). The n-3 docosapentaenoic acid (DPA): a new player in the n-3 long chain polyunsaturated fatty acid family. Biochimie.

