# Supplementary material for: Fads2 knockout mice reveal that ALA prevention of hepatic steatosis is dependent on delta-6 desaturase activity
Source: J Lipid Res. 2024 Sep 19;65(10):100642. doi: 10.1016/j.jlr.2024.100642 (PMC11526206; doi:10.1016/j.jlr.2024.100642)
Supplement: Supplemental Table S1 [file mmc1.docx]

Supplemental Table S1. Percentage fatty acid composition of lard, flax, and menhaden diets, as determined by gas chromatography.

| **Fatty Acid** | **LARD** | **FLAX** | **MENHADEN** |
| --- | --- | --- | --- |
| **14:0** | 1.38% ± 0.01 | 0.14% ± 0.01 | 11.45% ± 0.09 |
| **16:0** | 23.48% ± 0.05 | 5.45% ± 0.1 | 23.09% ± 0.15 |
| **16:1n7** | 1.66% ± 0.01 | 0.09% ± 0.12 | 16.98% ± 0.15 |
| **18:0** | 14.18% ± 0.19 | 4.25% ± 0.01 | 4.16% ± 0 |
| **18:1n9** | 36.02% ± 0.32 | 21.61% ± 0.03 | 7.06% ± 0.04 |
| **18:2n6** | 18.82% ± 0.09 | 17.55% ± 0.06 | 2.23% ± 0 |
| **18:3n3** | 1.38% ± 0.08 | 46.97% ± 0.19 | 2.34% ± 0 |
| **20:0** | 0.33% ± 0.03 | 0.54% ± 0.23 | 0.49% ± 0.05 |
| **20:4n6** | 2.52% ± 0.01 | 2.37% ± 0.06 | 1.7% ± 0 |
| **20:5n3** | 0% ± 0 | 0% ± 0 | 16.62% ± 0.13 |
| **22:0** | 0.14% ± 0.02 | 0.37% ± 0.07 | 0.43% ± 0.09 |
| **24:0** | 0.08% ± 0.12 | 0.27% ± 0.15 | 0% ± 0 |
| **22:6n3** | 0% ± 0 | 0.14% ± 0.2 | 13.01% ± 0.15 |
| **24:1n9** | 0% ± 0 | 0.24% ± 0.09 | 0.44% ± 0.01 |
| **Total**  **SFA** | 39.60% ± 0.35 | 11.03% ± 0.07 | 39.61% ± 0.1 |
| **Total MUFA** | 37.68% ± 0.33 | 21.94% ± 0.19 | 24.48% ± 0.19 |
| **Total N-3 PUFA** | 1.38% ± 0.08 | 47.11% ± 0 | 31.97% ± 0.28 |
| **Total N-6 PUFA** | 21.34% ± 0.1 | 19.93% ± 0.11 | 3.93% ± 0.01 |

All data is reported as mean ± standard deviation (SD).
