# Supplementary material for: Fads2 knockout mice reveal that ALA prevention of hepatic steatosis is dependent on delta-6 desaturase activity
Source: J Lipid Res. 2024 Sep 19;65(10):100642. doi: 10.1016/j.jlr.2024.100642 (PMC11526206; doi:10.1016/j.jlr.2024.100642)
Supplement: Supplemental Table S2 [file mmc2.docx]

Supplemental Table S2. Primer sequences for RT-qPCR.

|  | Forward | Reverse | Amplicon Size (bp) | Primer Efficiency (%) |
| --- | --- | --- | --- | --- |
| *Fads1* | AAC ATG CAC CCC CTC TTC TT | TGG TTG TAT GGC ATG TGC TT | 86 | 96.7% |
| *Fads2* | CCT GCT GAT TGG TGA GCT G | TGA AGT CCT CGG TGA TCT GA | 77 | 94.6% |
| *Dgat1* | GCC CCA TGC GTG ATT ATT | TCT GTC AGG GCA CCC ACT | 86 | 98.3% |
| *Dgat2* | GGC GCT ACT TCC GAG ACT AC | TGG TCA GCA GGT TGT GTG TC | 60 | 97.9% |
| *Agpat3* | TCA GGG TCA CGT CAT AGA TAG C | CTG CCC CCA CTC AAG TAC C | 103 | 96.9% |
| *Ppar-α* | AAC TGG ATG ACA GTG ACA TTT CC | CCC TCC TGC AAC TTC TCA AT | 103 | 98.8% |
| *Cpt1a* | GGC ATA AAC GCA GAG CAT TCC TG | CAG TGT CCA TCC TCT GAG TAG C | 110 | 100% |
| *Srepb1c* | ATC GGC GCG GAA GCT GTC GGG GTA GCG TC | ACT GTC TTG GTT GTT GAT GAG CTG GAG CAT | 116 | 100% |
| *Scd1* | GAG ACC TGA TAC CTA ACA CTC TGT CA | GAT GTG ATG TTT TCT TCT AGA CTT TCC | 79 | 97.7% |
| *Fasn* | CCA AAT CCA ACA TGG GAC A | TGC TCC AGG GAT AAC AGC A | 76 | 100% |
| *Acc* | GCG TCG GGT AGA TCC AGT T | CTC AGT GGG GCT TAG CTC TG | 70 | 100% |
| *Pck-1* | GGA GTA CCC ATT GAG GGT ATC AT | GCT GAG GGC TTC ATA GAC AAG | 75 | 98.6% |
| *Gk* | CTG GGG CCT TGG AAG GTG | TTT TGG CCT GTC CAT CCT GG | 96 | 100% |
| *Aqp9* | GCT GAA AGG CCA AAC AGT GG | TAG TGG TAG CGC TCA GCT TG | 87 | 94.1% |
| *18s* | CAA CTT CTT AGA GGG ACA AGT GG | CGG ACA TCT AAG GGC ATC AC | 74 | 96.4% |
