# Supplementary material for: Fads2 knockout mice reveal that ALA prevention of hepatic steatosis is dependent on delta-6 desaturase activity
Source: J Lipid Res. 2024 Sep 19;65(10):100642. doi: 10.1016/j.jlr.2024.100642 (PMC11526206; doi:10.1016/j.jlr.2024.100642)
Supplement: Supplemental Table S3 [file mmc3.docx]

Supplemental Table S3. Fatty acid content (nmol FA/g liver) from wildtype (WT) and *Fads2* knockout (KO) mice fed a lard, flax, or menhaden diet.

| Fatty Acid | | LARD | | FLAX | | | | MENHADEN | |  | |
| --- | --- | --- | --- | --- | --- | --- | --- | --- | --- | --- | --- |
|  |  | *WT* | *KO* | | *WT* | *KO* | *WT* | | *KO* | | *P_D_, P_G_, P_I_* |
| 14:0 | TAG | 5732.6 ± 513.3 | 5159.2 ± 1206.7 | | 2667.5 ± 776.5 | 2096.9 ± 298.6 | 2614.7 ± 456.7 | | 6979.7 ± 2631.5 | | *P_D_ = 0.0275, P_G_ = n.s., P_I_ = n.s.* |
|  | PL | 541.4 ± 36.9 | 666.2 ± 34.6 | | 626.2 ± 108.0 | 547.9 ± 54.6 | 777.9 ± 38.1 | | 854.0 ± 75.2 | | *P_D_ = 0.0012, P_G_ = n.s., P_I_ = n.s.* |
| 16:0 | TAG | 37639.6 ± 2753.5 | 30460.5 ± 6929.9 | | 14010.7 ± 4030.6 | 13739.8 ± 1895.7 | 12842.5 ± 1824.4 | | 25948.7 ± 7756.5 | | *P_D_ = 0.0002, P_G_ = n.s., P_I_ = n.s.* |
|  | PL | 4017.3 ± 328.7 | 4245.4 ± 295.2 | | 3272.9 ± 386.9 | 3366.4 ± 140.0 | 7663.7 ± 263.8 | | 8227.6 ± 622.8 | | *P_D_ <0.0001, P_G_ = n.s., P_I_ = n.s.* |
| 16:1n7 | TAG | ND | ND | | ND | ND | ND | | ND | | ND |
|  | PL | ND | ND | | ND | ND | ND | | ND | | ND |
| 18:0 | TAG | 9589.6 ± 1337.6 | 7999.6 ± 1682.2 | | 4540.4 ± 1239.6 | 4429.9 ± 787.2 | 2400.0 ± 440.6 | | 4397.4 ± 1152.3 | | *P_D_ <0.0001, P_G_ = n.s., P_I_ = n.s.* |
|  | PL | 33121.1 ± 2230.6 | 33849.9 ± 2304.1 | | 30983.8 ± 1193.0 | 34038.5 ± 1257.1 | 27702.2 ± 936.3 | | 32204.9 ± 1336.7 | | *P_D_ = n.s., P_G_ = n.s., P_I_ = n.s.* |
| 18:1n9 | TAG | 245714.5 ± 29365.8 | 227460.8 ± 58094.1 | | 70530.0 ± 18658.6 | 96638.6 ± 155550.1 | 28984.3 ± 4255.6 | | 48302.4 ± 13180.6 | | *P_D_ <0.0001, P_G_ = n.s., P_I_ = n.s.* |
|  | PL | 18042.5 ± 806.6 | 24855.7 ± 1258.7* | | 12902.5 ± 791.0 | 19490.6 ± 1046.8* | 11919.3 ± 658.3 | | 12214.1 ± 1382.8 | | *P_D_ <0.0001, P_G_ <0.0001, P_I_ = 0.0027* |
| 18:2n6 | TAG | 49258.8 ± 7519.4 | 42294.7 ± 6101.7 | | 12016.4 ± 2429.2 | 22974.0 ± 4503.9 | 3357.3 ± 424.6 | | 5045.3 ± 1403.2 | | *P_D_ <0.0001, P_G_ = n.s., P_I_ = n.s.* |
|  | PL | 19570.6 ± 833.7 | 34498.4 ± 2480.3* | | 18324.2 ± 1265.3 | 31212.9 ± 1195.8* | 5426.3 ± 362.1 | | 6283.8 ± 264.4 | | *P_D_ <0.0001, P_G_ <0.0001, P_I_ <0.0001* |
| 18:3n3 | TAG | 4788.5 ± 1101.7 | 6417.6 ± 1761.6 | | 19948.3 ± 2577.6 | 51049.3 ± 6033.9* | 1548.5 ± 181.8 | | 1924.2 ± 304.8 | | *P_D_ <0.0001, P_G_ <0.0001, P_I_ <0.0001* |
|  | PL | 361.1 ± 45.3 | 561.2 ± 54.0 | | 3735.2 ± 358.3 | 7149.1 ± 335.2* | 402.1 ± 19.8 | | 468.2 ± 45.9 | | *P_D_ <0.0001, P_G_ <0.0001, P_I_ <0.0001* |
| 20:0 | TAG | 995.6 ± 107.8 | 807.7 ± 222.2 | | 312.8 ± 54.0 | 422.4 ± 49.9 | 274.0 ± 30.0 | | 334.6 ± 42.6 | | *P_D_ <0.0001, P_G_ = n.s., P_I_ = n.s.* |
|  | PL | 285.0 ± 25.8 | 312.3 ± 26.6 | | 341.5 ± 30.4 | 285.1 ± 13.5 | 367.1 ± 57.0 | | 503.7 ± 40.3 | | *P_D_ = 0.0006, P_G_ = n.s., P_I_ = 0.0388* |
| 20:4n6 | TAG | 5626.0 ± 1331.1 | 2826.471 ± 478.0 | | 1511.8 ± 542.5 | 1520.0 ± 342.1 | 668.2 ± 76.7 | | 797.4 ± 96.2 | | *P_D_ <0.0001, P_G_ = n.s., P_I_ = n.s.* |
|  | PL | 51177.9 ± 2746.8 | 55271.8 ± 3731.3 | | 33024.9 ± 1199.0 | 52309.7 ± 1527.6* | 14517.9 ± 947.4 | | 16111.6 ± 920.2 | | *P_D_ <0.0001, P_G_ <0.0001, P_I_ = 0.0005* |
| 20:5n3 | TAG | 600.5 ± 90.0 | 31.1 ± 24.8 | | 2856.0 ± 1000.0 | 167.4 ± 41.1* | 6354.8 ± 937.4 | | 5870.5 ± 971.8 | | *P_D_ <0.0001, P_G_ = 0.0326, P_I_ = n.s.* |
|  | PL | 398.3 ± 54.6 | ND | | 8807.4 ± 1167.2 | 224.8 ± 26.6* | 17256.4 ± 1277.6 | | 16349.5 ± 1535.3 | | *P_D_ <0.0001, P_G_ = 0.0002, P_I_ = 0.0002* |
| 22:0 | TAG | 228.2 ± 24.0 | 204.9 ± 27.3 | | 116.3 ± 8.8 | 171.9 ± 27.6 | 113.6 ± 9.9 | | 147.6 ± 15.4 | | *P_D_ = 0.0001, P_G_ = n.s., P_I_ = n.s.* |
|  | PL | 263.0 ± 26.7 | 287.4 ± 34.5 | | 196.8 ± 23.2 | 184.5 ± 20.7 | 201.0 ± 17.8 | | 224.7 ± 29.7 | | *P_D_ = 0.0058, P_G_ = n.s., P_I_ = n.s.* |
| 22:6n3 | TAG | 2258.6 ± 799.5 | 26.4 ± 19.1 | | 3843.5 ± 1078.7 | 268.6 ± 105.8 | 12693.1 ± 1788.4 | | 14229.8 ± 1320.4 | | *P_D_ <0.0001, P_G_ = 0.0283, P_I_ = n.s.* |
|  | PL | 13811.0 ± 809.1 | 1502.6 ± 157.8* | | 18802.5 ± 1284.2 | 535.7 ± 53.8* | 39418.8 ± 1224.6 | | 46775.7 ± 2062.6* | | *P_D_ <0.0001, P_G_ <0.0001, P_I_ <0.0001* |
| 24:0 | TAG | ND | ND | | ND | ND | ND | | ND | | ND |
|  | PL | 0.20 ± 0.20 | ND | | ND | 84.78 ± 42.44 | ND | | ND | | ND |
| 24:1 | TAG | ND | ND | | ND | 249.4 ± 63.7 | ND | | ND | | ND |
|  | PL | ND | ND | | ND | ND | ND | | ND | | ND |
| Total SFA | TAG | 217865.1 ± 24533.9 | 180659.9 ± 49054.0 | | 73823.3 ± 18905.6 | 73370.4 ± 9394.8 | 54172.6 ± 7573.1 | | 103217.3 ± 24680.5 | | *P_D_ <0.0001, P_G_ = n.s., P_I_ = n.s.* |
|  | PL | 82424.05 ± 4571.8 | 84509.4 ± 4828.0 | | 79555.0 ± 2619.7 | 75548.8 ± 1488.1 | 91165.8 ± 2229.6 | | 101453.6 ± 4355.4 | | *P_D_ <0.0001, P_G_ = n.s., P_I_ = n.s.* |
| Total MUFA | TAG | 245714.5 ± 29365.8 | 227479.6 ± 58092.2 | | 70530.1 ± 18658.6 | 96888.1 ± 15583.7 | 28984.3 ± 4255.6 | | 48302.4 ± 13180.6 | | *P_D_ <0.0001, P_G_ = n.s., P_I_ = n.s.* |
|  | PL | 18042.5 ± 806.6 | 24855.7 ± 1258.7* | | 12902.5 ± 791.0 | 19490.6 ± 1046.8* | 11919.3 ± 658.3 | | 12214.1 ± 1382.8 | | *P_D_ <0.0001, P_G_ <0.0001, P_I_ = 0.0027* |
| Total PUFA | TAG | 62532.4 ± 10453.7 | 54170.3 ± 7187.1 | | 76363.8 ± 32394.9 | 92938.2 ± 16646.8 | 27484.9 ± 3589.8 | | 27867.3 ± 3725.5 | | *P_D_ = 0.0347, P_G_ = n.s., P_I_ = n.s.* |
|  | PL | 85318.84 ± 4077.1 | 91672.1 ± 5946.4 | | 82694.2 ± 3772.2 | 91666.0 ± 2539.9 | 76765.5 ± 1743.0 | | 84209.9 ± 3238.1 | | *P_D_ = n.s., P_G_ = 0.0224, P_I_ = n.s.* |
| Total TAG | | 526112.0 ± 59494.5 | 462309.8 ± 112656.8 | | 220717.1 ± 67158.3 | 263196.7 ± 41321.3 | 110641.8 ± 14624.5 | | 179387.0 ± 43865.2 | | *P_D_ <0.0001, P_G_ =n.s., P_I_ = n.s.* |
| Total PL | | 185785.3 ± 9256.5 | 201037.2 ± 11646.3 | | 175151.8 ± 6397.6 | 186705.4 ± 4648.4 | 179850.5 ± 4471.9 | | 197877.58 ± 8948.7 | | *P_D_ = n.s., P_G_ = 0.0303, P_I_ = n.s.* |

ND: not detected or below 0.01 nmol FA/g liver; TAG: triacylglycerol; PL: phospholipid. * represents a significant genotype difference between diet groups (*P < 0.05*). All data is reported as mean ± standard error mean (SEM).
